# Management of Unilateral Vocal Fold Paralysis after Thyroid Surgery with Injection Laryngoplasty: State of Art Review

**DOI:** 10.3389/fsurg.2022.876228

**Published:** 2022-04-06

**Authors:** Li-Jen Liao, Chi-Te Wang

**Affiliations:** ^1^ Department of Otolaryngology Head and Neck Surgery, Far Eastern Memorial Hospital, Taipei, Taiwan; ^2^ Department of Electrical Engineering, Yuan Ze University, Taoyuan, Taiwan; ^3^ Department of Special Education, University Of Taipei, Taipei, Taiwan

**Keywords:** vocal cord paralysis, injection laryngoplasty, dysphonia, larynx, review

## Abstract

**Background:**

Unilateral vocal fold paralysis (UVFP) after thyroid surgery often leads to significant morbidity including dysphonia, dysphagia, and aspiration. Injection laryngoplasty (IL) is an effective treatment of UVFP with numerous readily available materials. However, few studies focus on IL for UVFP following thyroidectomy.

**Objectives:**

This review aims to critically review current literature to determine the timing, materials, methods and outcomes of IL for UVFP after thyroid surgery.

**Type of Review:**

Literature review.

**Methods:**

A literature review was performed using the Pubmed, Medline and EMBASE databases. All relevant articles published in English addressing the effect of IL in post thyroid surgery related UVFP were analyzed. Studies using IL for UVFP of multiple etiologies were excluded. Meta-analysis was conducted using fixed and random effect model.

**Results:**

Five original studies were identified, including 214 patients received IL for UVFP following thyroid surgery. Two studies injected autologous fat via direct suspension laryngoscope under general anesthesia, while the other 3 studies injected polyacrylamide, hyaluronic acid, and polymethyl methacrylate from cricothyroid membrane under local anesthesia. All 5 studies reported improved voice outcomes of IL for post-thyroidectomy UVFP. Meta-analysis showed MPT increased for 3.18 s (95% CI: 2.40–3.96, fix effect model) after IL. Another common acoustic parameter, jitter (%) also improved for 1.46 (95% CI: 0.73–2.19, random effects model) after IL for post-thyroidectomy UVFP.

**Conclusions:**

This review supported that IL can improve the voice outcome for post-thyroidectomy UVFP. Autologous fat remains a good augmentation material with a potential longer lasting effect. More research and long-term surveys are needed to document the safety and longevity of other synthetic materials.

## Background

The synonymous term of unilateral vocal fold paralysis (UVFP) includes vocal cord palsy, vagal paralysis and recurrent laryngeal neuropathy. Iatrogenic injury is now the prevailing etiologies for UVFP (1) and thyroid surgery related recurrent laryngeal nerve injury is one of the most common cause for iatrogenic UVFP. The an average incidence of transient and permanent UVFP following thyroid surgeries were 9.8 and 2.3%, respectively (2). UVFP often leads to significant morbidity that may include dysphonia, dysphagia, aspiration, or even pneumonia after thyroid surgery, especially in elder patients (3). Several modalities had been introduced for post-thyroidectomy UVFP, e.g., voice therapy, medialization thyroplasty, and injection laryngoplasty (IL).

The first IL was reported by Dr. Bruening in 1911 using liquid paraffin (4). Unfortunately, this material is not tissue compatible and resulted in chronic granuloma formation and material extrusion (5). Later on, IL using Teflon paste was re-introduced in 1960’s (6). Although short-term effectiveness was satisfactory, Teflon paste was gradually noted to cause serious long-term sequel, i.e., Teflon granuloma, owing to profound foreign body reaction (7). After the frustrating experience with paraffin and Teflon (8), subsequent study shifted to more histologically compatible materials, i.e. homologous and autologous collagen (9–11), and bovine / porcine collagen (12, 13). Since 2000s, other synthetic compatible materials had been introduced to clinical use during IL (14), e.g. carboxymethylcellulose (15), hyaluronic acid (13, 16), and calcium hydroxylappatite (17).

Compared with other treatment modalities for post-thyroidectomy UVFP, IL has several advantages. IL can be performed in the office under local anesthesia. Real-time feedback of voice improvement can also be conducted. Most-importantly, patient did not need another open-surgery (e.g. thyroplasty) to correct UVFP resulting from prior thyroid surgeries. From our clinical experience, IL is well tolerated in the office and most patients exhibit stable hemodynamics throughout the procedure (18). Although IL is effective for UVFP, most of the existing studies included a mixture of different etiologies of UVFP (e.g., iatrogenic, neoplastic, idiopathic). Only a few studies focused on IL in the management of post-thyroidectomy related UVFP. Accordingly, we conducted this literature review to summarize the state of art practice and evidence in this specific clinical scenario.

## Material and Method

A literature review was performed using the Pubmed, Medline and EMBASE database. The following keywords and MeSH Terms were applied: vocal fold palsy OR vocal cord palsy AND injection therapy. All relevant articles published in English addressing the effect of injection laryngoplasty in UVFP were reviewed. We limit the literatures into IL for UVFP after thyroidectomy. Studies including multiple etiologies of UVFP were excluded. We evaluated the risk of bias in recruited studies using Risk of Bias in Non-Randomized Studies of Interventions (ROBINS-I) (19).

Retrieved information include the number of patients, injected material, injection approach, and treatment outcomes before and after injection laryngoplasty. Owing to different reporting timeline across each studies, outcomes of the longest follow-up period was selected for subsequent meta-analysis (R software, version 4.1.2, with packages for meta-analysis (20). We adapted results from either fixed or random effect model for statistical inference based on the significance of potential heterogeneity among the recruited research.

## Results

**Figure 1:**
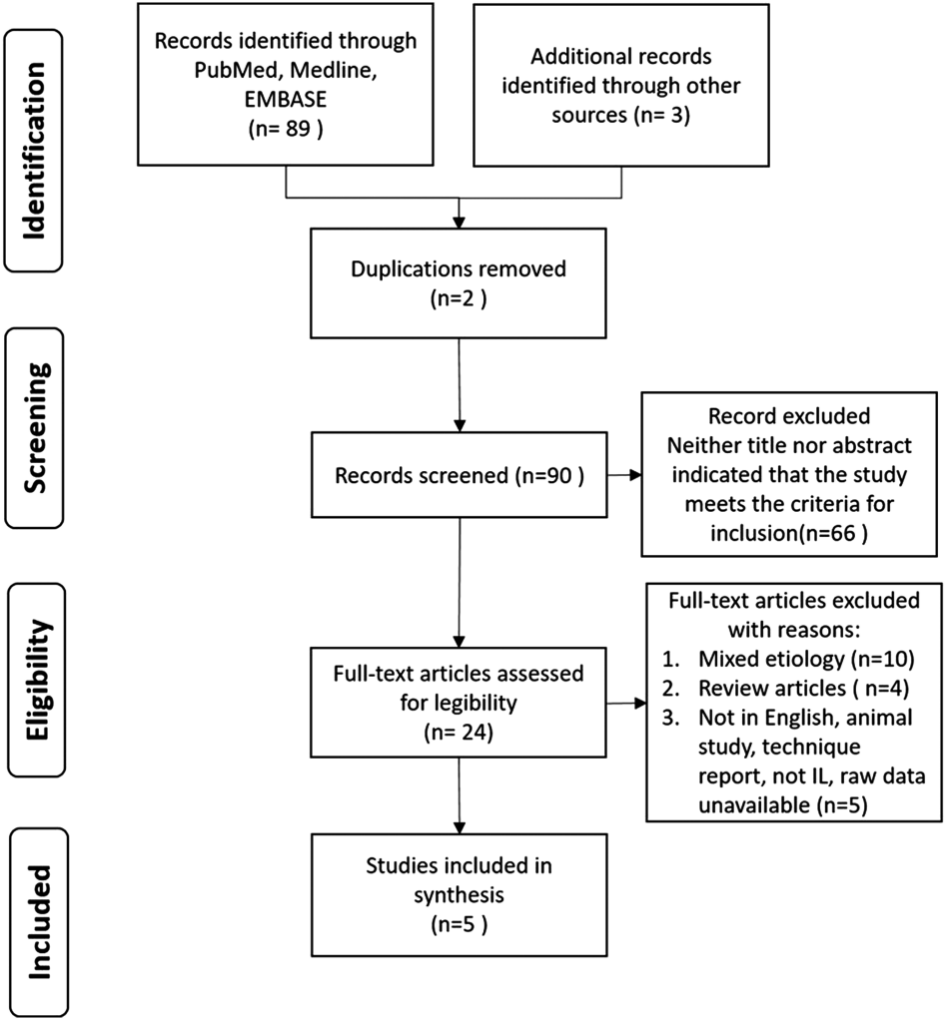
PRISMA flow diagram depicts the process of literature searching.

The flow chart of the study selection process is shown in Figure 1. We identified 5 original articles summarized in Table 1. Totally 214 patients received IL after thyroid surgery related UVFP were reported. Fang et al. (21) reported acoustic outcomes of 27 patients with autologous fat injection and follow-up the residual fat volume with 3-dimensional imaging. The mean residual fat volume remained consistent after 26-month follow-up. The maximal phonation time (MPT), s/z ratio, jitter, and noise-to-harmonic ratio (NHR) were significantly improved during follow-up.

**Table 1:** Summary for the literatures focus on the management of UVFP after thyroid surgery with IL.

	Authors	Main findings
1	Fang et al. (21)	27 patients with autologous **fat injection** MPT, s/z ratio, jitter, and harmonic-to-noise ratio were significantly improved. Mean residual fat volume remained consistent after 26-month follow-up.
2	Lee et al. (22)	34 patients with **PAAG (PAAG (Aquamid®) and Hyaluronic acid (Rofilan®) injection** Acoustic and perceptual parameters (overall GBRAS), MPT, jitter, and shimmer, voice handicap index, and grades of mucosal waves and glottic closure were significantly improved and remained stable over 6 months
3	Jang et al. (23)	55 patients (24 early, 31 late injection) with **PMMA (polymethyl methacrylate, ArteSense™)** and early voice rehabilitation All tested voice parameters were significantly improved in both the early and late groups. The amount of voice improvement was significantly larger in the early injection group, especially jitter % (*P* = 0.02) and shimmer % (*P* = 0.03).
4	Chun et al. (24)	25 patients received IL using **hyaluronic acid**, comparing with 23 patients received voice therapy Greater improvement in UVFP patients who underwent IL then voice therapy
5.	Lin et al. (25)	73 patients underwent **autologous fat injection** Gender and age may stand as significant categories on analysis voice indicators

*GBRAS, grade of hoarseness, roughness, breathiness, asthenia, and strain.*

S/Z ratio, the maximal length when pronouncing “S”, divided by the maximal length when pronouncing “Z”.

Lee et al. (22) reported 34 patients received polyacrylamide hydrogel (PAAG (Aquamid®) for permanent UVFP and Hyaluronic acid (Rofilan®) for temporary UVFP after thyroidectomy. Acoustic and perceptual parameters (GRBAS), MPT, jitter, and shimmer, voice handicap index, and grades of mucosal waves and glottic closure were all significantly improved after the injection and remained stable over 6 months.

Jang et al. (23) reported 55 patients injected with PMMA (polymethyl methacrylate, ArteSense™, a relatively long-lasting injectable substance for soft-tissue augmentation) for UVFP after thyroidectomy. The authors further divided these patients into 24 early injection (within 3 months between IL and thyroid surgery) and 31 late injection (IL at longer than 3 months after thyroidectomy). All of the measured objective and subjective voice parameters were significantly improved in both the early and late groups. The degree of voice improvement was significantly larger in the early injection group, especially jitter % (*P* = 0.02) and shimmer % (*P* = 0.03) improvement.

Chun et al. (24) reported 25 patients of post-thyroidectomy with aspiration symptoms receiving injection laryngoplasty using hyaluronic acid (Rofilan), and another 23 patients without aspiration receiving only voice therapy. They found greater improvement in thyroidectomy-related voice questionnaire, GRBAS scale, jitter, shimmer and NHR in patients who underwent injection laryngoplasty comparing to voice therapy alone.

Lin et al. (25) reported 73 patients underwent autologous fat injection for UVFP after thyroid surgery. They reported a significant improvement of multi-dimensional voice parameters 1 year after lipoinjection. This study also found that patients under 60 years old presented better improvement of MPT then the other patient older than 60 years. BMI did not alter post-operative voice parameters, whereas sex may present differently upon acoustic analysis.

The numerical outcomes were summarized in Table 2. We also evaluated the risk of bias in the recruited studies using ROBINS-I tool as shown in Table 3. Most of recruited studies reveal low risk of bias, 2 studies have moderate concern of missing data bias due to short follow-up time; one study has moderate concern of bias in selection of the main reported result (lack of MPT outcomes); and the other one study had moderate bias in participant selection (vague description of inclusion criteria).

**Table 2:** Compare the effects of ILs for post thyroid surgery UVFP with different injection material.

	Pre- treatment	3–6 month	12-month	Post- treatment
**MPT (seconds)**				
Fat (Fang et al. (21))	**4.9 ** **± ** **2.9**	9.3 ± 3.1	9.9 ± 2.5	**10 ** **± ** **3.2**
PAAG &HA (Lee et al. (22))	**4.7 ** **± ** **2.9**		8.8 ± 4.5	**7.8 ** **± ** **5.4**
TVFP	7.8 ± 4.9	12.1 ± 4.4		
PVFP	4.3 ± 1.9	8.2 ± 3.6		
PMMA (Jang et al. (23))				
Early IL	**5.60 ** **± ** **3.19**	**7.93 ** **± ** **3.29**		
Late IL	**5.41 ** **± ** **3.68**	**7.61 ** **± ** **3.77**		
Fat (Lin et al. (25))	**5.95 ** **± ** **4.15**		8.77 ± 4.92	
**Jitter (%)**				
Fat (Fang et al. (21))	**3.1 ** **± ** **1.7**	1.2 ± 0.6	1.2 ± 0.7	**1.0 ** **± ** **0.4**
PAAG &HA (Lee et al. (22))	**2.9 ** **± ** **1.1**		3.0 ± 2.7	**2.2 ** **± ** **0.6**
TVFP	3.8 ± 1.0	1.3 ± 0.3		
PVFP	3.6 ± 2.6	2.7 ± 0.8		
PMMA (Jang et al. (23))				
Early IL	**5.12 ** **± ** **4.81**	**2.22 ** **± ** **1.80**		
Late IL	**3.89 ** **± ** **2.34**	**3.10 ** **± ** **4.18**		
HA (Chun et al. (24))	**3.36 ** **± ** **2.05**	2.12 ± 1.38	**1.85 ** **± ** **1.23**	

*MPT, Maximal phonation time; PAAG, PAAG (Aquamid®); HA, Hyaluronic acid (Rofilan®); TVFP, transient vocal fold paralysis; PVFP, permanent vocal fold paralysis; IL, injection laryngoplasty. The bold values are used in meta-analysis.*

**Table 3:** Evaluating the risk of bias in recruited studies using ROBINS-I (19).

Publications	D1	D2	D3	D4	D5	D6	D7
Fang et al. (21)	**L**	**L**	**L**	**L**	**L**	**L**	**L**
Lee et al. (22)	**L**	**L**	**L**	**L**	**L**	**L**	**L**
Jang et al. (23)	**L**	**L**	**L**	**L**	**M**	**L**	**L**
Chun et al. (24)	**L**	**L**	**L**	**L**	**L**	**L**	**M**
Lin et al. (25)	**L**	**M**	**L**	**L**	**M**	**L**	**L**

*Domains included in ROBINS-I.*

*D1: Bias due to confounding.*

*D2: Bias in selection of participants into the study.*

*D3: Bias in classification of interventions.*

*D4: Bias due to deviations from intended interventions.*

*D5: Bias due to missing data.*

*D6: Bias in measurement of outcomes.*

*D7: Bias in selection of the reported result.*

*Judgement: Low risk of bias: **L**; Moderate risk of bias: **M**; Serious risk of bias: **S**; Critical risk of bias: C; No information: NA.*

**Figure 2:**
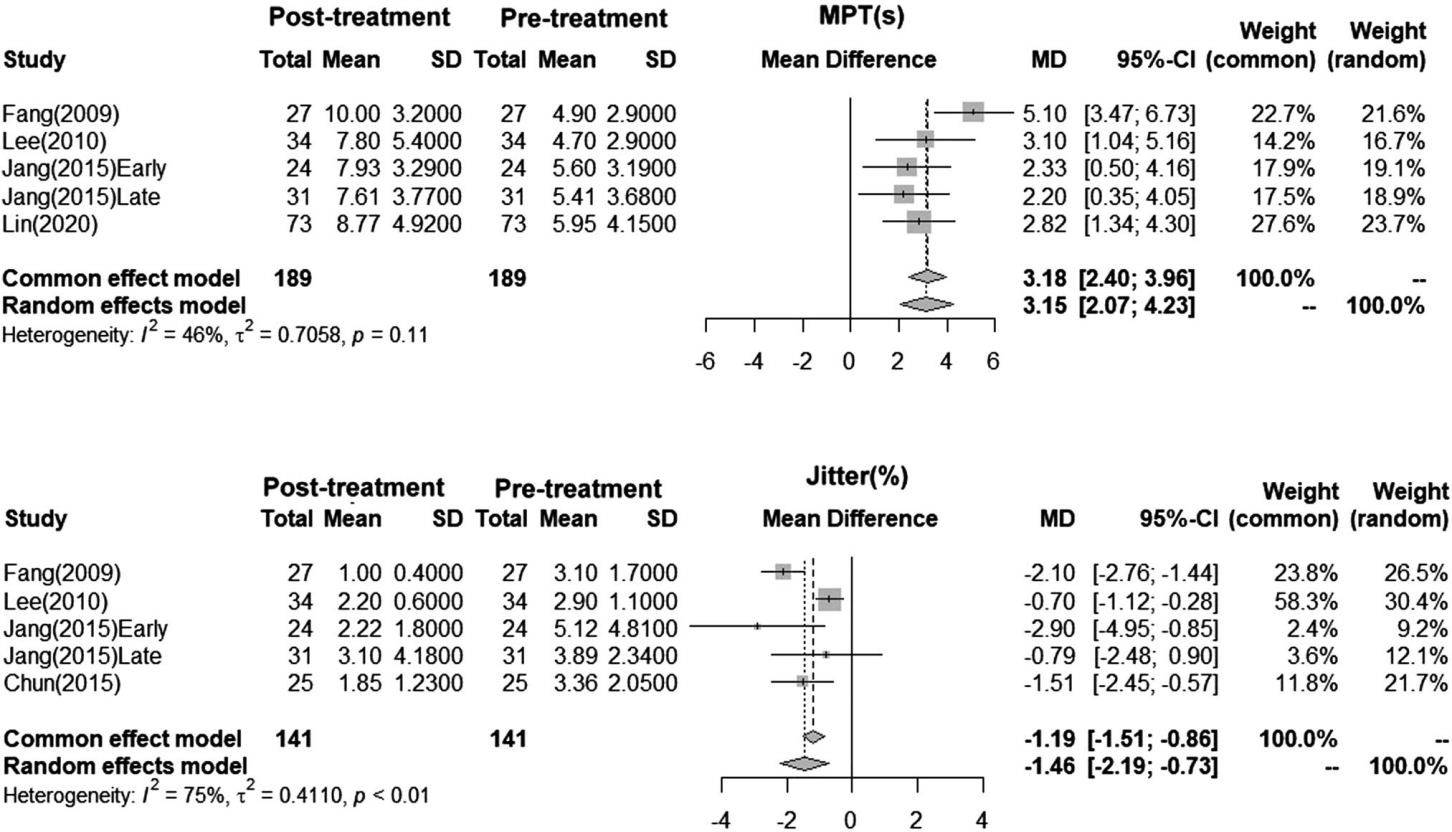
Forest plots show the results of meta-analysis from the included research. Fixed effect (i.e., common effect) model was selected for statistical inference when heterogeneity between research was non-significant, whereas random effects model was applied in presence of significant heterogeneity.

We combined the result of these studies for subsequent meta-analysis. The first outcome parameter iS MPT, reported among 4 studies. Because the heterogeneity test showed non-significance (I square = 46%, *p* = 0.11), we adapted the results of fixed effect model (Figure 2). Meta-analysis showed an increased MPT of 3.18s (95% CI: 2.40–3.96) after IL. The second outcome parameter was jitter (%), which was also reported in 4 studies. Considering significant heterogeneity among the recruited research (I square = 75%, *p* < 0.01), we adapted results of random effect model which showed an improvement of 1.46 (95% CI: 0.73–2.19).

## Discussion

UVFP often leads to significant morbidity that may include dysphonia, swallowing problems and aspiration after thyroid surgery. Conservative treatment via voice therapy may ameliorate part of the symptoms of UVFP (26, 27). For patients not responding to voice therapy, surgical correction include injection laryngoplasty, medialization thyroplasty, arytenoid adduction and reinnervation of recurrent laryngeal nerve (28). Chen et al. (29) conduct a meta-analysis for management of UVFP, they recommend absorbable material injection laryngoplasty during the first year and reinnervation after 12 months. According to another systematic review for UVFP management, earlier IL is suggested to decrease the necessary of subsequent medialization thyroplasty (30). Considering IL for UVFP after thyroid surgery, Jang et al. (23) reported that the amount of voice improvement was significantly larger in the early injection group, especially in jitter (%) (*P* = 0.02) and shimmer (%) (*P* = 0.03). Therefore, earlier IL is suggested for post thyroid surgery related UVFP.

Despite continual reports show that IL is effective and are available for treatment of UVFP (16, 28–31), most of the published literature mixed with different etiologies of UVFP. Considering the prevalence of thyroid neoplasm and the high incidence of thyroidectomy-related UVFP (2), this review specifically retain only original papers reporting IL for UVFP after thyroid surgeries. Considering the potential heterogeneity when pooling the effectiveness of IL, we adapted the results from either fixed effect or random effect model. Our literatures review supported that IL is an effective management for post-thyroidectomy related UVFP. Further meta-analysis showed IL could increase MPT for 3.18 (2.40–3.96) seconds and decrease Jitter (%) for 1.46 (0.73–2.19), both results were statistically significant (Figure 2).

Different injection material for IL were noticed in this literature review and may be further divided into temporary versus permanent intentions. Temporary material as hyaluronic acid and permanent material as autologous fat are the most common injection materials for IL. In the report by Fang et al, CT scan showed that injected autologous fat remained in situ with a mean interval of 26 ± 13 months after initial IL. The parameters from acoustic analyses also revealed stable results after 12 months, indicating that autologous fat may be a potential long-term filler. Similar results had also been reported by Umeno et al. (32), which showed that only a minimum patients (<5%) needs secondary IL following autologous fat injection. Nevertheless, controversial findings from other studies showed higher failure rates ranged from 30% to 41%, and patients may need further revision fat injection after 12 to 24 months (33, 34). Possible explanations for such a great diversity include different donor site of adipose tissues, harvesting techniques (e.g., liposuction vs. mincing), additive insulin, centrifugation, size of the injection needle, and pressurized instrument (35–38).

**Figure 3:**
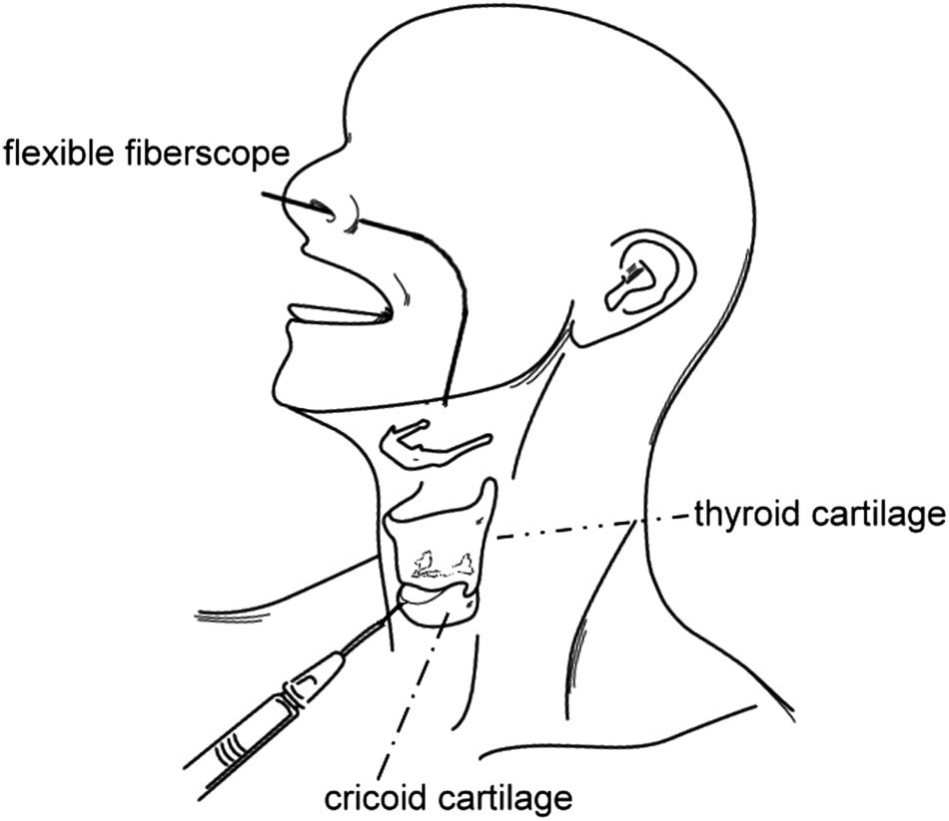
IL performed under local anesthesia with flexible fiberscope guidance via cricothyroid membrane.

Another advantage of IL is that it could be performed under local anesthesia with multiple injection routes (39). In this review, 3 article performed transcutaneous injection route from cricothyoid membrane (Figure 3) (22–24). Other methods include ultrasound guided (40) or EMG guided (41, 42) injection. Otherwise, IL may also be performed under routine general anesthesia with direct laryngoscope suspension, similar to the 2 studies using autologous fat for IL in this review (21, 25). With regarding to the voice outcome, in our opinion, no technique is superior to other approaches. The choice of guiding and injection technique depends on the patient’s preference and the operator’s experience.

The long-term effect of IL remains undetermined. The recruited studies did not report the percentage of patients who need repeated IL or laryngeal framework surgery. Limited by varying reported parameters (2 studies using VHI-30 (22, 23) while another study use VHI-10 (25), we cannot perform a meta-analysis using patient-reported outcomes in this study. In addition, some of these studies were conducted retrospectively via chart review (23, 25) and may present some degree of bias. Further prospective study is still necessary to confirm the longer effect of IL for post-thyroidectomy related UVFP.

## Conclusion

This review supported that IL could improve the voice outcome for post-thyroidectomy UVFP. Autologous fat remains a good augmentation material with a potential longer lasting effect. More research and long-term survey might be needed to document the safety and longevity of other synthetic materials.

## Data Availability

The original contributions presented in the study are included in the article/supplementary material, further inquiries can be directed to the corresponding author’s.
